# Application of Structured Light System Technique for Authentication of Wooden Panel Paintings

**DOI:** 10.3390/s16060881

**Published:** 2016-06-14

**Authors:** Fernando Buchón-Moragues, José María Bravo, Marcelino Ferri, Javier Redondo, Juan Vicente Sánchez-Pérez

**Affiliations:** 1 Departamento de Ingeniería Catográfica, Geodesia y Fotogrametría, Universitat Politécnica de Valéncia, Camino de Vera s/n, 46022 Valencia, Spain; fbuchon@upv.es; 2 Centro de Tecnologías Físicas, Acústica, Materiales y Astrofísica, División Acústica, Universitat Politécnica de Valéncia, Camino de Vera s/n, 46022 Valencia, Spain; jobrapla@upv.es; 3 Instituto de Investigación para la Gestión Integrada de zonas Costeras, Universitat Politécnica de Valéncia, Paranimf 1, Grao de Gandia, 46730 Valencia, Spain; mferri@upv.es (M.F.); fredondo@upv.es (J.R.)

**Keywords:** pictorial artworks authentication, pictorial artworks cataloging, non-destructive testing, structured light system technique

## Abstract

This paper presents a new application of photogrammetric techniques for protecting cultural heritage. The accuracy of the method and the fact that it can be used to carry out different tests without contact between the sample and the instruments can make this technique very useful for authenticating and cataloging artworks. The application focuses on the field of pictorial artworks, and wooden panel paintings in particular. In these works, the orography formed by the brushstrokes can be easily digitalized using a photogrammetric technique, called Structured Light System, with submillimeter accuracy. Thus, some of the physical characteristics of the brushstrokes, like minimum and maximum heights or slopes become a fingerprint of the painting. We explain in detail the general principles of the Structured Light System Technique and the specific characteristics of the commercial set-up used in this work. Some experiments are carried out on a sample painted by us to check the accuracy limits of the technique and to propose some tests that can help to stablish a methodology for authentication purposes. Finally, some preliminary results obtained on a real pictorial artwork are presented, providing geometrical information of its metric features as an example of the possibilities of this application.

## 1. Introduction

Photogrammetry can be defined as a set of techniques to determine the geometric properties of objects from photographic images. Photogrammetry belongs to a group of methods usually called Non-Destructive Testing (NDT) that allow different tests to be performed on objects without damaging them, avoiding direct contact between the sample and the instruments used. One of the most reliable and accurate photogrammetric techniques developed in recent decades is known as the Structured Light System Technique (SLST) [1,2]. SLST allows the three-dimensional (3D) surface reconstruction of objects with very high accuracy, and nowadays is being applied in a variety of fields including medical applications such as dermatology [3], dentistry [4] and orthopedics [5], and industrial uses [6] for example, in the textile industry [7].

Among all the possible applications, photogrammetry in general and SLST in particular have been revealed in recent years as powerful methods to help protect and document cultural heritage, and especially to obtain details or certain pieces of the facades of historic buildings [8]. SLST has been successfully applied in the scanning of sculptures due to its high precision and relatively low cost [9]. SLST has also been used in the field of archeology to recreate details of excavated surfaces and associated artifacts at two Middle Paleolithic sites in southwest France [10]. In the field of underwater archeology, SLST has been used in a submerged environment with different turbidity degrees to provide whole-field 3D reconstructions of underwater archaeological pieces [11]. Sometimes SLST is used together with a laser scanner because it complements the 3D models obtained with the scanner offering greater precision.

In the field of pictorial artworks, passive multispectral and multimodal imagery techniques have been used for years to highlight and understand conservation and restoration tasks, so that intervention plans can be designed to prevent further deterioration of artworks. An active method based on the projection of a grid of parallel lines using SLST onto the surface of a twelfth-century masterpiece has been developed to study deformations of panel paintings correlated with environmental changes and has provided a topographic map [12]. SLST has been also used in the scanning of the “Adoration of the Magi” by Leonardo da Vinci, giving rise to a study documenting the spatial deformation suffered by paintings on wood [13]. Coded SLST is a mature technology and offers high resolution for analyzing the relief in paintings [14]. In 2011, an interdisciplinary team of photogrammetrists, art-historians, restorers and experts in non-destructive diagnostic techniques made a comprehensive review of multispectral and multimodal imagery techniques. A particularly interesting study uses 3D imaging and photogrammetric methods to evaluate deformation in various important paintings, as well as the topography of the brushstrokes and detachment of the upper paint layer, providing detailed maps of the deterioration [15]. Furthermore, a very high-resolution automated measurement prototype for digitizing paintings has been developed and checked on ancient paintings. The prototype consists of a specially designed frame system for secure fastening of the painting without touching its surface, based on SLST with no Infrared or Ultraviolet emissions [16]. 

Following this line, in this paper we present an application of SLST devoted to obtaining 3-Dimensional Surface Digital Models (3DSDMs) of wooden panel paintings. These models provide geometrical information about the metric features of the paintings, which can be considered unique digital fingerprints. Specifically, the 3DSDMs allow the brushstrokes that form the orography of the painting to be vectorized with submillimeter accuracy. Thus, SLST can be used for authentication tasks to detect any change in any stroke if this change is within the sensitivity of the measuring instrument. Although SLST can be applied using different commercial systems, while obtaining preliminary results we selected one specific type formed by a low cost hardware coupled with free software, which can therefore be widely used.

The paper is organized as follows: In Section 2 we explain the basic principles of the SLST and the specific details of the particular set-up used. In Section 3, some experimental results are shown, checking the performance of the particular set-up selected and proposing some tests that can help to establish a methodology for the use of SLST for authentication purposes. Finally, the last section contains concluding remarks, and a summary of the most important results.

## 2. Measurement Methodology and Experimental Set-up

In the 1980s some authors [17] developed a measurement system based on the projection of different patterns of light on an object, which are captured by a photographic or video camera that records the pattern shape of the object from which a three-dimensional model is then obtained. These kinds of projected patterns can be codified in different ways, according to the ranking proposed by Salvi *et al.* [18]: Time-Multiplexing, Spatial Neighborhood or Direct Coding. The SLST proposed here is included in the Time-Multiplexing codification, in particular with the projection mode of binary code. The SLST set-up has two main elements: (i) a video camera, which uses Charge-Coupled Device (CCD) sensors to convert incident light into analogous voltage, quantifying it in bits values (maybe even a webcam could be used); (ii) a light source, which projects the pattern of a binary light on the object to detect its orography.

**Figure 1:**
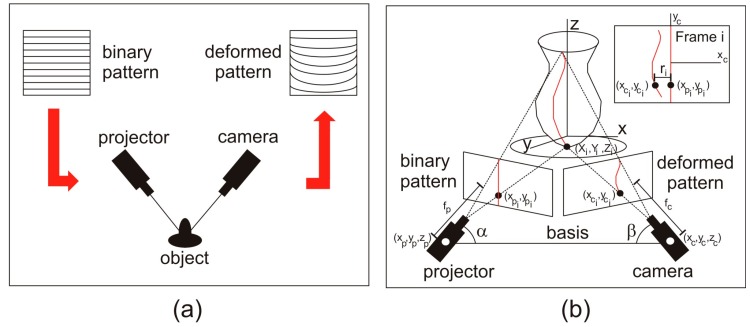
(**a**) Photogrammetric triangulation for the binary code used in this work; (**b**) Outline of the process of obtaining 3-Dimensional Surface Digital Models (3DSDM) of objects using the Structured Light System Technique (SLST).

Geometric principles of the SLST are based on the projection of a series of predetermined binary patterns on the object to be measured, and subsequent analysis of their deformation. Thus, a photogrammetric triangulation is produced between the projected image (binary pattern), the object, and the image captured by a camera (deformed pattern). In Figure 1a one can see a scheme of the process for the case of the binary code used in this work, showing the deformation produced on this binary pattern captured by the camera to be projected onto the object. To measure this deformation, the geometry between the different elements: camera–projector distance (called basis), angles between the main directions of both the camera and the projector with the basis (α,β), and the coordinates of the centers of the camera and projector have to be determined in the calibration of the system. The calibration will be carried out by performing a set of measurements on a panel with a dot matrix known (calibration panel), applying the principles of space resection where each point of the calibration panel is determined by two lines: one defined by the projection center of the projector and the point of the image of the pattern projected, and the other that includes the projection center of the camera and the point of the captured image.

With all these parameters, a system of equations is posed, in which the following parameters are involved and related: (i) coordinates of the center of the camera (x_c_, y_c_, z_c_) and projector (x_p_, y_p_, z_p_); (ii) coordinates of the points of the projected (x_pi_, y_pi_) and captured (deformed) images (x_ci_, y_ci_); (iii) camera and projector focal lengths (f_c_, f_p_); (iv) rotations for the orientation of the camera (κ_c_, ϕ_c_, ω_c_) and projector (κ_p_, ϕ_p_, ω_p_); and (v) terrain coordinates of each point of the measured object (X_i_, Y_i_, Z_i_). The system defines the geometric structure of the measuring set-up and, depending on the phase (calibration or measurement), some of these parameters act as unknowns or data. The system of equations formed is usually called “bundle adjustment” and its resolution is based on the use of least square method. A comprehensive development of the approach and resolution of the method can be seen in [19]. Note that any change in the position of the different elements of the set-up will require recalibration.

For a better understanding, it is possible to simplify the methodology taking into account that each one of the projected binary patterns is formed by different linear elements, which deform when projected on an uneven surface. Considering only a single linear element (stripe) and analyzing its deformation when projected on the object, as one can see in Figure 1b, each deformation value (r_i_) can be obtained by a simple comparison with the original pattern. This value is called parallax, and provides the depth of each point, allowing obtaining the tridimensional coordinates and the 3DSDM of the object. This process is outlined in Figure 1b.

Among the different set-ups available for obtaining 3DSDMs by means of SLST, we have chosen a low cost hardware called DAVID SLS-1 [20]. A description of its characteristics is given below.

**Figure 2:**
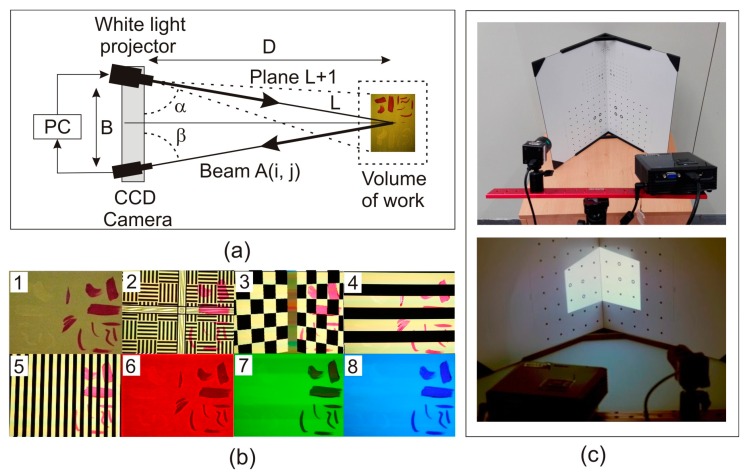
(**a**) Schematic explanation of the photogrammetric method used; (**b**) Capture images of the 3-dimensional digital model of the wooden panel painting; (2b1) sample used in the first experiment; (2b2–2b3) two views of the calibration of the system using a predefined standard; (2b4–2b5) Two views of the measurement procedure with two of the 58 binary patterns used; (2b6–2b8) Red-Green-Blue (RGB) color model fields capture to obtain the texture of the sample; (**c**) top: DAVID SLS-1 instrumental which includes the projector, the camera and one of the calibration templates used; bottom: a view of the calibration process, where one of the calibration templates made of glass with reticles of coded points of different sizes can be seen.

DAVID SLS-1 is a registered trademark based on photogrammetric triangulation applied to SLST. Figure 2a shows the operating scheme used to obtain the tridimensional (3D) coordinates. This set-up uses a light generator ACER K11+, which projects energy as a binary pattern of light on the object to be measured, and a video camera with a resolution of 1280 × 960 pixels with a 12 mm COMPUTAR lens that records images of the different patterns projected. DAVID SLS-1 has a default accuracies range from 0.05 mm to 0.5 mm depending on several parameters of system configuration such as: size of the scanned sample, calibration pattern used, distance from the camera to the projector and distance from the scanner to the sample. The noise generated at each measurement can be reduced using the high number of patterns allowed by the set-up (58 in quality mode, 26 in defect mode and 22 in velocity mode). DAVID SLS-1 has several calibration templates made of glass with reticles of coded points of different sizes, which are placed squarely allowing calibration of the system and determination of projector-camera angles and distance and the calibration parameters of the camera like the size of the image and the radial and tangential distortions. This procedure generates a calibration file that can be used in several measurements if the measurement system remains unchanged. 

The shooting process is carried out as we have explained before: each band defined by the binary pattern (striped pattern) generates a distorted line (stripe) adapted to the object to be measured, which is collected on a photogram. Once the images have been captured, and taking as reference the pattern projected on the sample, the image coordinates of the edge points of the stripes captured by the camera can be easily obtained from the coordinate differences between the reference pattern and the one projected onto the sample (parallaxes r_i_). Thus, the 3D coordinates of each point identified in the images will be obtained in a coordinate system defined by the projector-camera set in the previous calibration. 

Equation (1) represents one of the relationships between the terrain coordinates (the 3D coordinates of each point of the sample) to be obtained (X_i_, Y_i_, Z_i_) with the parameters defined in the calibration process of the measurement set-up:

(1) $$\left[\begin{array}{c} X_{i}\\ Y_{i}\\ Z_{i} \end{array}\right]\;=\;\frac{B}{f\;\cot\alpha -x}\;\left[\begin{array}{c} x_{ci}\\ y_{ci}\\ f \end{array}\right]$$

where B is the basis, defined as the distance between the projection centers of both the camera and the projector; α is the angle between the basis and the direction of the projected stripe; f is the camera focal length, and (x_ci_, y_ci_) are the image coordinates in the camera. This equation is a standard and easy resolution of a triangle using trigonometry, on which the triangulation method is based. For further details see reference [21]. The working process is developed along the following steps:

(i) Setting and calibration of the parts that form the DAVID SLS-1 system: This set-up is portable, so it has the advantage of lightness. However, due to its portability, the distance between the projector and the camera (measurement basis) must always be set depending on the size of the sample. Once the basis is defined, the system must be calibrated by performing a first measurement using a previously calibrated standard. The reason for this first measurement, as we have explained above, is to determine the parameters of the system configuration which define the photogrammetric triangulation, and then the coordinates of the sample are obtained in a metric coordinate system (Figure 2(b2,b3)).
(ii) Measurements: Once the sample is in the area to be captured by the camera, it is illuminated by the light projector and a set of 58 different binary patterns are projected and captured by the camera. From each of these patterns a set of terrain coordinates is obtained and by combining all the coordinates obtained from the calculation of each pattern, a 3DSDM of the surface of the sample is achieved. If the sample is too large to be measured in one shot then a registration process for the unification of all 3DSDM shots should be performed (Figure 2(b4,b5)).
(iii) Texture: To apply a realistic texture on the measured 3DSDM, three shots are taken with the camera, each corresponding to the Red-Green-Blue (RGB) color model fields (Figure 2(b6–b8)). In this work, we have used the texture of the models only to enable adequate understanding of the process.

Two different views of this measurement procedure, where the DAVID SLS-1 system can be observed, are presented in Figure 2c. As we commented above, the optimal accuracy in the measurements is 0.05 mm in absolute position in determining a point in space, and can be considered as a sphere of uncertainty. Consequently, any variation in the brushstrokes from the original sample that is equal to or greater than this distance can be detected and taken as reference the 3DSDM previously obtained.

## 3. Results and Discussion

**Figure 3:**
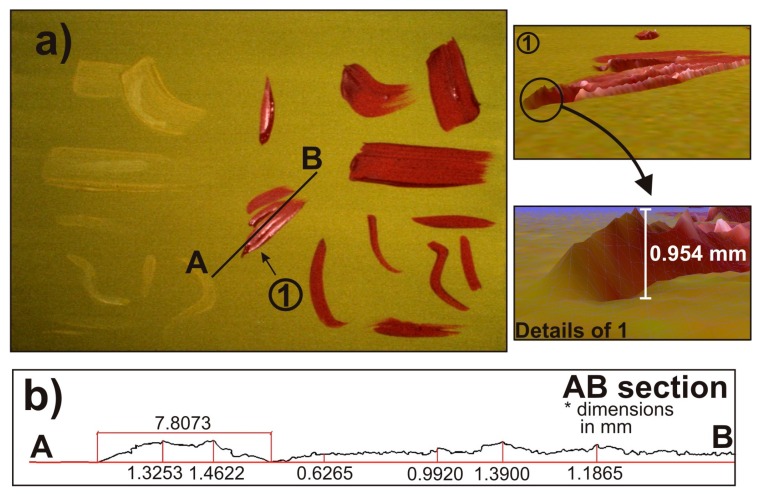
(**a**) Painting test sample. Detail of the 3DSDM of the painting (1) with the relief of the brushstrokes. Note the precision of the method in the inset, where a detail of a height of the brushstroke is 0.954 mm; (**b**) Geometrical details of an AB section of the brushstroke marked in (**a**). One can see the precision in the measurements of the 3DSDM of the sample, which can help to authenticate pictorial artworks.

To analyze the limits of the application of SLST in the field of cataloging and authentication of wooden panel paintings, we carried out a test series. First of all, we painted a flat board with a layer of yellow primer. Next, we added several brushstrokes imitating the ones that can be found in a real painting, with two different colors, yellow and red. The idea was to obtain a 3DSDM of the sample to measure, among other things, some geometric characteristics of the brushstrokes regardless of the color used. This sample is shown in Figure 3a.

After preparing the sample, we obtained the 3DSMD of the painting using DAVID SLS-1 as explained in Section 2. Figure 3a shows some of the results, where one can observe a specific view of the 3DSDM carried out (marked as 1), observed under the point of view marked by the arrow in Figure 3a. To determine the accuracy of the SLST used, we measured the height of the end of the brushstroke shown in Figure 3a. The inset shows the general shape of this particular brushstroke with its height, which is 0.954 mm. Figure 3b shows an A-B cross-section of the sample, which is marked in Figure 3a. One can see different heights of this section, as well as the width of the nearest part of the brushstroke to point A with a high degree of accuracy.

The SLST enabled us to determine the height of several details of the painting and analyze stroke style. The high resolution of SLST allows in-depth analysis of the orography of the paints with the usual commercial software used in 3DSDM. Thus, obtaining a 3DSDM of pictorial artworks offers an opportunity to analyze them from a new perspective. With this tool, the cataloging and authentication of wooden panel paintings can become a very accurate process.

The cataloging process makes a preliminary analysis of the author’s pictorial. This process involves studying the physical characteristics of the strokes of several of the artist’s paintings, as if it were a 3DSDM of a plot of land, determining some physical facts about the different strokes such as the slope or the position of the top of the strokes.

Furthermore, the proposed methodology can be interesting for authentication purposes if the original painting has been previously scanned, due to the high accuracy of the 3DSDM. It does not seem possible to falsify a painting if the accuracy of the digital model is under 0.05 mm. It is only possible to determine if the sample is authentic by scanning the sample under suspicion and compare it with the previous 3DSDM done. Thus, the digital model is a sort of fingerprint of the analyzed artwork practically impossible to reproduce. 

**Figure 4:**
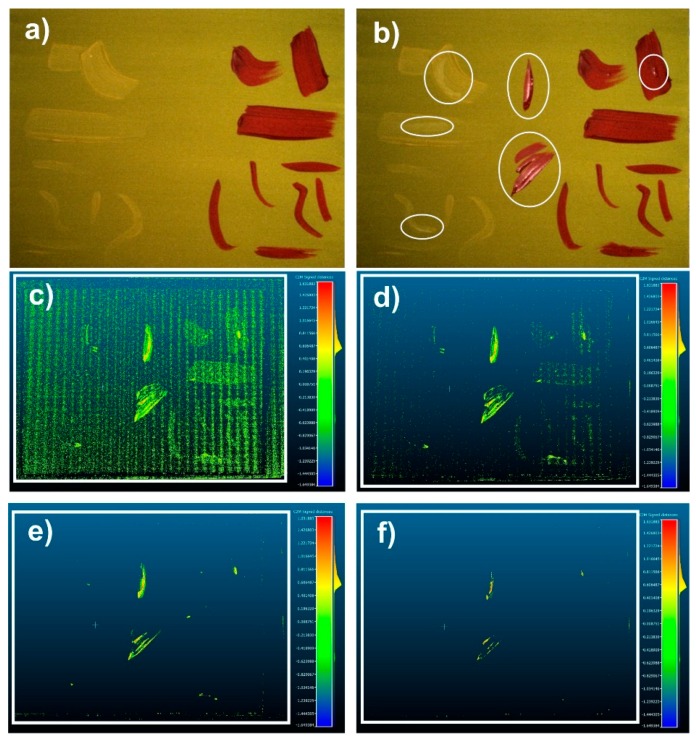
Initial (**a**) and modified (**b**) samples. One can see the new added brushstrokes; (**c**–**f**) Different horizontal cuts of the painting at different heights: (**c**) 0.05 mm; (**d**) 0.1 mm; (**e**) 0.2; (**f**) 0.5 mm.

As we have explained, comparison between different scans carried out on different dates is important. To describe the way to do that, we have developed a test: on the previous sample used in Figure 3a and shown again in Figure 4a, new smooth brushstrokes are painted (see Figure 4b) in order to vary the starting sample. The goal of this test is to evaluate the precision of the method used, detecting these new strokes. One can observe that some of the new strokes are clearly visible in the modified sample, while others are not so obvious because they are hidden in the primer layer or in previous strokes. Using a free software called “cloud compare” [22], it seems possible to overlap both 3DSDM (the initial model and the modified one) to obtain the differences between them.

The comparison methodology used by “cloud compare” between 3DSDMs is based on the “Iterative Closest Point (ICP) point to mesh” algorithm [23]. In order to avoid possible misalignment between the two meshes (starting and modified), we made some marks in the external *passé partout* joined to the sample, while the measurement system remains unchanged throughout the measurement process. These marks, which appear in both 3DSDMs, help us to align both meshes successfully. The variations in height for different cuts between the two considered samples are visualized in green from Figure 4c to Figure 4f. Figure 4c shows a cut in the range 0–+0.05 mm, where we are at the limits of measurement noise and no conclusions can be drawn. In Figure 4d, the cut is between the mesh elements which vary in height between +0.05 mm and +0.1 mm. Here, noise values are scarce and the new brushstrokes begin to be clearly defined. However, some preexisting brushstrokes can still be seen in the figure (bottom right area), probably due to its red hue that can present some distortion points generating high levels of noise. The results of the cut between +0.1 mm and 0.2 mm are represented in Figure 4e, where only the thicker new brushstrokes are clearly shown. Finally, in Figure 4f, one can see the top of these thick brushstrokes, corresponding to a height over +0.5 mm. Note that in these last two figures, noise has virtually disappeared. Therefore the height of the new strokes can be measured and their shape can be determined with accuracy, showing that even minimal changes to the initial painting can be detected with SLST.

**Figure 5:**
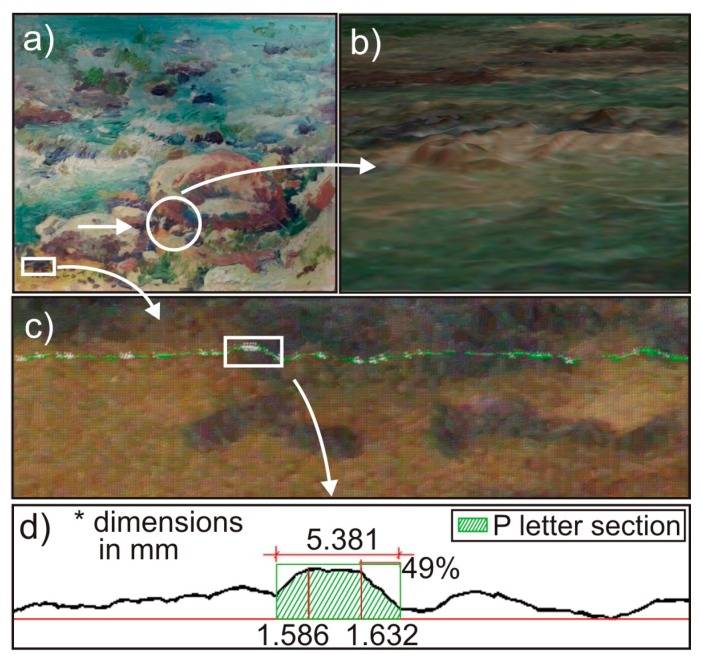
Photogrammetry with DAVID SLS-1 of a real artwork. (**a**) Photo of the wooden panel painting by J. Peris Aragó (0.20 m × 0.25 m); (**b**) Detail of the 3DSDM; (**c**) Part of the signature on the painting, showing detail of the letter “P” (Peris Aragó) and the cross section in green in (**d**) are shown; (**d**) Cross section of part of the signature of the painting marked in (**c**). One can see all the geometrical characteristics of the section of the letter “P” in the signature (width, heights and slope of the right side).

Finally, in order to apply this technique to a real case, we obtained the 3DSDM of a wooden panel painting by the Spanish painter, José Peris Aragó, carrying out a preliminary analysis of its metric features. The analyzed painting belongs to a private collection, and it has been ceded by the owner for this study. José Peris Aragó, born in Alboraya (Valencia), was a painter who lived in the twentieth century. The impressionistic character of some of his works was decisive in choosing the painting, because its reliefs can be analyzed accurately using the method described here. A photograph of the painting is shown in Figure 5a. A detail of the painting, marked with a circle in Figure 5a, can be seen in Figure 4b. One can observe the orography of this part of the artwork, where the geometrical information on the brushstrokes can be measured with a high degree of accuracy. In Figure 5c,d, we carried out a preliminary geometrical data collection of a part of the signature on the painting, marked with a white square in Figure 5a. A cross-section of part of the signature was obtained, shown in green in Figure 5c, where the letter “P” (Peris) has been transversally cut. In Figure 5d we show the details of this cross-section, where numerical data, focused on the cut of the letter “P” were determined. Thus, two different heights, the width of the section and the slope of the right side of the letter “P” are shown. These details, with the precision of the method (0.05 mm), representing a sort of fingerprint of the painting, and can help in the processes of authentication or cataloging simply by obtaining different 3DSDMs over time, as we have explained throughout this work.

## 4. Conclusions

This work highlights a new application of a photogrammetric technique called Structured Light System Technique (SLST) in the field of protection of cultural heritage. SLST enables obtaining 3-Dimensional Surface Digital Models (3DSDMs), which provide geometrical information of the surface of pictorial artworks with high accuracy. This digital format represents a sort of fingerprint of the artwork and can be used for several applications, such as authentication or cataloging, among others.

The use of a low cost set-up coupled with low cost software, and the rapidity in collecting the 3DSDM data are some of the advantages of the specific commercial SLST used. On the other hand, the restrictions in using this technique when the heights of the brushstrokes are under the accuracy requirements of the set-up, or the fact that the 3DSDM only provides information of the surface of the pictorial artwork and not on its inner layers, can be quoted as possible disadvantages.

In this article, we present some preliminary results, proposing some tests that could be the foundations of a methodology for authentication and cataloging of pictorial artworks. To verify their authenticity at any given moment, obtaining of a new 3DSDM should suffice, performing an overlay analysis of both models and determining the geometrical differences between the two. For cataloging purposes, the development of a statistical analysis to determine the physical characteristics of the brushstrokes of a painter in a given period (height, slope, *etc.*) is proposed. However, more studies must be carried out in order to define a robust methodology for using SLST for authentication or cataloging purposes.
